# Theoretical exploration of inherent electronic, structural, mechanical, thermoelectric, and thermophysical response of KRu_4_Z_12_ (Z = As_12_, Sb_12_) filled skutterudite materials

**DOI:** 10.1039/d3ra05546a

**Published:** 2023-09-19

**Authors:** Poorva Nayak, Pankakaj Srivastava, Dinesh C. Gupta

**Affiliations:** a Condensed Matter Theory Group, School of Studies in Physics, Jiwaji University Gwalior India 474 011 poorvanayak11@gmail.com sosfizix@gmail.com; b Atal Bihari Vajpayee Indian Institute of Information Technology and Management Gwalior India 474015

## Abstract

Using the density functional theory methodology, we have thoroughly examined KRu_4_As_12_ and KRu_4_Sb_12_ skutterudites, including their structural, electronic, mechanical, transport, and thermodynamic properties. First and foremost, using the Birch–Murnaghan equation of state, the structural stability has been calculated in terms of their total ground state and cohesive energies. With the use of the approximation approaches GGA and GGA + mBJ, the electrical structure and density of the states reveal their metallic nature. This demonstration predicts the dominant ferromagnetic spin configuration of materials by considering their electronic behavior and magnetic interactions. The ductile behavior of these alloys is also addressed by their mechanical qualities, which indicate how they might be used in engineering and industrial settings. Moreover, the semi-classical Boltzmann transport theory has been employed to examine the Seebeck coefficient as well as the electric and thermal conductivities. The general tendency of these compounds demonstrates their various potential uses as electrode materials. The quasi-harmonic Debye approximation is a method used to analyze the stability of a system under high pressures and accounts for the temperature dependency of thermodynamics. It combines the quasi-harmonic approximation, which considers the anharmonicity of vibrations, with the Debye model, which describes the vibrational modes of a solid. This approach allows for a more accurate representation of the system's behavior at different temperatures and pressures. By implementing this approximation, researchers can gain insights into the stability and thermodynamic properties of materials under extreme conditions.

## Abstract 2

The filled skutterudite KRu_4_Z_12_ (Z = As_12_, Sb_12_) crystallizes in the unique BCC structure with space group *Im*3̄ and contains 17 atoms per unit cell. The K atom takes the atomic position of (0 0 0), Ru atoms are positioned at (0.25 0.25 0.25), and Sb & As atoms are located at (0, 0.35, 0.16).

## Graphical abstract

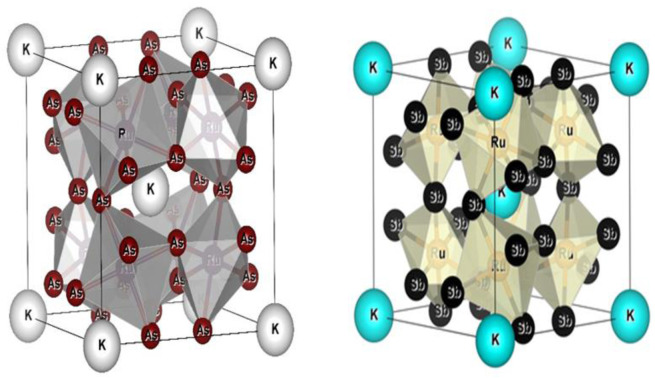

## 1. Introduction

CoSb_3_, with its thermal stability and high Seebeck coefficient at room temperature, emerges as an attractive candidate for mid-temperature thermoelectric (TE) applications. CoSb_3_ is a compound composed of cobalt (Co) and antimony (Sb) elements, considered less toxic compared to other compounds and is relatively abundant in nature.^1^ Unfilled and doped thermoelectric (TE) skutterudites have garnered considerable attention, especially in the last decade, offering advantages like low cost due to the absence of rare earths, ease of processing, and the ability to be synthesized as both n- and p-type TE materials.^2^ In recent decades, there has been renewed interest in developing novel enhanced thermoelectric substances for cooling and power generation applications, driven by consumption habits and growing energy demand. Reports indicate that nearly 66% of primary energy is wasted as heat, with only 33% being used for actual work.^3^ Thermoelectric materials provide a solution by directly converting waste heat into electricity through the Seebeck effect. Solid-state thermoelectric devices, acting as thermoelectric generators, can generate a voltage potential by applying a temperature difference, or as refrigerators, functioning as micro-coolers.

Thermoelectric generators (TEGs) can convert waste heat generated by various sources, such as solar irradiation, heat generated in car exhaust, or industrial processes, into useable electricity. Moreover, these thermoelectric materials retain mechanical stability, high reliability, long-term equipment, and low environmental impact, offering practical advantages without the need for moving parts.^4,5^ Similarly, skutterudite compounds represent a promising class of materials with excellent thermoelectric properties at high temperatures. In thermoelectric materials, the figure of merit (*ZT*) is a key parameter that determines performance. It is calculated using the equation (*ZT* = *S*^2^*σT*/*κ*), where *S* represents the Seebeck coefficient, *σ* is the electrical conductivity, *κ* denotes the thermal conductivity, and *T* is the absolute temperature. These properties are interconnected and play a crucial role in determining the efficiency of thermoelectric materials. Researchers aim to enhance the performance of thermoelectric materials for various applications such as waste heat recovery and solid-state cooling by optimizing these factors. For a material to exhibit good thermoelectric performance, it must satisfy several requirements. Not only should its Seebeck coefficient and electrical conductivity be high, but it should also possess a lower thermal conductivity (*κ*) value. A material's thermal conductivity (*κ*) is determined by the sum of electronic (*κ*_e_) and lattice (*κ*_l_) components. Low *κ* and high *S* are required to achieve high *ZT*. Despite this, the band structure and scattering mechanisms are tightly coupled and mutually constrain these parameters, making it challenging to separate the electrical and thermal performance parameters. According to the Weidmann–Franz law, *κ*_e_ = *LσT*, where *κ*_e_ is closely related to *σ*, while *κ*_l_ is unrelated to the electrical properties. Researchers are trying to implement various optimization methods to reduce the *κ*_l_ of materials as an effective way to improve *ZT* values.^6–13^ The efficiency of a thermoelectric (TE) device hinge significantly on the choice of p- and n-type TE materials. Recent research has explored various TE materials, investigating their electrical and thermal properties.^14^ The article titled “vibrational and structural properties of the RFe_4_Sb_12_ (R = Na, K, Ca, Sr, Ba) filled skutterudites” discusses the vibrational and structural characteristics of filled skutterudites with different R elements (Na, K, Ca, Sr, Ba) through experimental and theoretical analysis.^15^ “High spin polarization in the ferromagnetic filled skutterudites KFe_4_Sb_12_ and NaFe_4_Sb_12_” explores the spin polarization properties of the ferromagnetic filled skutterudites KFe_4_Sb_12_ and NaFe_4_Sb_12_, investigating their electronic structure and magnetic properties using experimental techniques and theoretical calculations. Both compounds exhibit high spin polarization, making them promising candidates for spintronic applications.^16^ “Insightful analysis of magneto-electronic, mechanical, and thermophysical properties of novel filled skutterudites LiFe_4_X_12_ (X = As, Sb) through *ab initio* calculations” explores the properties of filled skutterudites LiFe_4_X_12_ (X = As, Sb) using *ab initio* calculations, focusing on magneto-electronic, mechanical, and thermophysical properties.^17^ “High-temperature electrical and thermal transport properties of fully filled skutterudites RFe_4_Sb_12_ (R = Ca, Sr, Ba, La, Ce, Pr, Nd, Eu, and Yb)” by Qiu *et al.*^18^ studied the high-temperature electrical and thermal transport properties of fully filled skutterudites RFe_4_Sb_12_ (R = Ca, Sr, Ba, La, Ce, Pr, Nd, Eu, and Yb). The filled skutterudite materials NaFe_4_Sb_12_ and KFe_4_Sb_12_ exhibit itinerant electron ferromagnetism characterized by high spin polarization.^19^ Conversely, the alkaline-earth-filled skutterudites, specifically AFe_4_Sb_12_ (A = Ca, Sr, Ba), manifest properties of nearly ferromagnetic systems.^20–23^

## 2. Computational methodology

The present calculations used the density functional theory calculation implemented in the WIEN2k code.^24^ The code is highly reliable and precise, thus providing better accuracy of results. In the present set of calculations, we have performed the exact computation. The calculation process begins with the simple generalized gradient approximation (GGA)^25^ to verify the intimate electronic structure and the density of states of KRu_4_As_12_ and KRu_4_Sb_12_. The main drawback of GGA is that it underestimates the electronic structure, especially in systems containing d/f electrons. Therefore, in response to this issue, we have adopted a modified Becke–Johnson (mBJ)^26^ to handle the exchange-correlation function. The calculations have been further extended by adopting the *R*_MT_*K*_MAX_ = 6.0, which controls the interstitial atomic size. The term “*R*_MT_” represents the smallest muffin-tin radius, and “*K*_MAX_” represents the maximum reciprocal lattice vector used in the plane wave expansion. The potential and charge density non-spherical contributions to muffin-tin (MT) spheres were expanded to *l*_max_ = 10, and the convergence criteria for energy and charge were set to 10^−4^ Ry and 10^−4^ eV. The tetrahedral method and a *k*-mesh of 1000 points in the Brillion zones (BZ) have been adopted. We have calculated the elastic properties by cubic elastic code.^27^ In addition to this, we have calculated the thermodynamic properties based on a quasi-harmonic Debye model using Gibbs2 code.^28^ The calculation of transport properties, such as the Seebeck coefficient (*S*), electrical conductivity (*σ*), thermal conductivity (*κ*), and power factor (PF), has been carried out by using the BoltzTraP code^29,30^ under constant relaxation time approximation.

## 3. Results and discussions

### 3.1 Structural stability

**Fig. 1:**
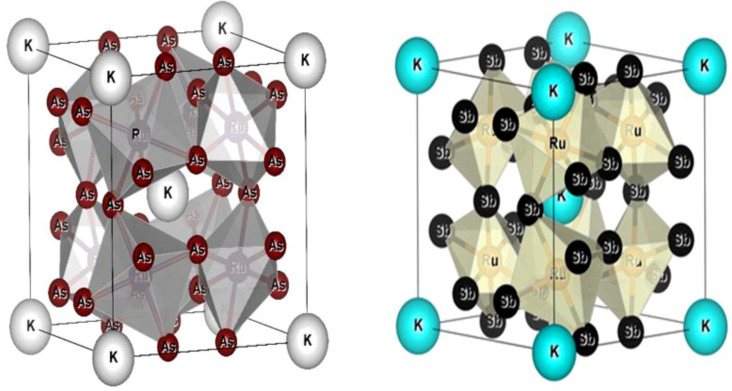
Molecular crystal structure of filled skutterudite KRu_4_As_12_ and KRu_4_Sb_12_.

**Fig. 2:**
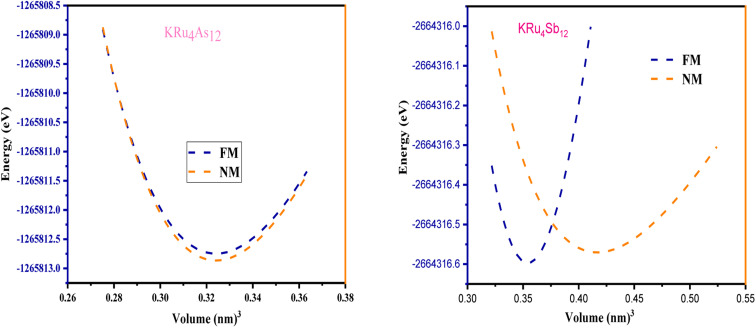
The variation of total energy (*E* in eV) with unit cell volume for KRu_4_Sb_12_ and KRu_4_As_12_.

All the selected compounds of the family adopt the CoAs_3_ type skutterudite structure, filled by alkaline earth metal atoms potassium (K), and based on one primitive cell per formula unit. In the early period, Oftedal described the structure of CoAs_3_ in 1928 (ref. 31) as a cubic structure with 32 atoms corresponding to the *Im*3̄ space group. However, the primitive unit cell contains seventeen atoms of three different types. The unit cell is composed of eight cubes of the transition metal (T = Ru) occupying the 8c sites (1/4, 1/4, 1/4), and 6 of these cubes are filled with square planar rectangles of the pnictogen (*P*_n_ = As, Sb) occupying the 24g (0, *y*, *z*) sites. The generic chemical formula for the filled skutterudites is MT_4_X_12_. The “filler” atom M consists of Ca, Sr, Ba, Hf, La, Nd, and Sm. Additionally, the transition metals T can be Fe, Ru, Rh, Os, while the pnictogens consist of P, As, and Sb. The rare earth atoms are positioned at the origin with coordinates (0, 0, 0), the transition metal is positioned at (0.25, 0.25, 0.25), and the pnictogen is positioned at (0, 0.35, 0.16) with varying parameters depending on the chemical composition. In the unit cell, the remaining two voids (2a at (0, 0, 0) or 1/2, 1/2, 1/2) can be filled by atoms whose ionic radii are smaller than the cage. An alkaline atom resides in position (2a) (0, 0, 0), four T (Ru) atoms at position (8c) (1/4, 1/4, 1/4), and twelve (pnictogen) X atoms at position (24g) (0, *y*, *z*). In the [T_4_X_12_] polyanion, *y* and *z* inner coordinates indicate the relative positions of X atoms. As a result, the structure of these alkaline-filled skutterudites is defined by one lattice parameter, *a* (nm), and two inner coordinates (*y* and *z*). It is commonly referred to as the half of the unit cell, T_4_Pn_12_, which has 88 valence electrons and is isoelectronic. The bond in the skutterudites is mainly covalent, as the distance between transition metal atoms is too great to form a bond. The interaction occurs between Pn–Pn (the pnictogen atom), forming Pn_4_ rings, and T–Pn bonds (metallic atom utilizing the pnictogen). The valence electron arrangement of the pnictogen atoms is ns^2^np^3^, each with 5 electron bands.^32^ The calculated total energies are plotted in Fig. 2 as a function of the unit cell volume for KRu_4_Sb_12_ and KRu_4_As_12_. We fit the total energy *versus* unit cell volume curve to Murnaghan's equation of state (EOS) to optimize both the compounds with two methods, ferromagnetic (FM) and non-magnetic (NM) phases. In addition to this, specific ground state properties such as volume (nm^3^), bulk modulus *B*_0_ (in GPa), its pressure derivative *B*′, and the minimum energy at the equilibrium *E*_0_ (in eV) parameters have been obtained and collected in Table 1. Fig. 1 shows the crystal structure of KRu_4_Sb_12_ and KRu_4_As_12_.

**Table 1:** Estimated lattice constant (*a* in nm), volume (*V* in nm^3^) bulk modulus (*B* in GPa), its pressure derivative *B*′, minimum energy (*E*_0_ in eV), and the cohesive energy (*E*_coh_ in eV per atom) for KRu_4_Sb_12_ and KRu_4_As_12_ compounds

Materials	Phase	*a*	*V*	*B*	*B*′	*E*_0_	*E*_coh_
KRu_4_As_12_	FM	0.865	0.3240	117.95	5.20	−1 265 182.75	4.02
	NM	0.865	0.3136	111.85	5.20	−1 247 467.64	
KRu_4_Sb_12_	FM	0.945	0.4227	92.09	4.85	−12 600 316.60	3.57
	NM	0.945	0.4167	91.65	5.05	−12 417 695.51	
LiFe_4_As_12_	FM	8.33 (ref. 17)	—	107.10	4.78	−64 463.72	4.54
	NM	—	—	—	—	−64 463.69	
LiFe_4_Sb_12_	FM	9.24 (ref. 17)		101.09	4.90	−165 804.41	3.95
	NM	—	—	—	—	−165 804.43	
KFe_4_As_12_	—	9.19 (ref. 35 and 16)	—	—	—	—	—
NaFe_4_As_12_	—	9.17 (ref. 36 and 37)	—	—	—	—	—
SrFe_4_Sb_12_	FM	9.26 (ref. 38 and 39)	—	123.3	2.87	−17 993.35	—
	NM	—	—	—	—	—	—
CoSb_3_	FM	9.03 (ref. 41)	1918.02	1117.7	4.63	−65 414.147	—

The assessment of chemical stability and the potential experimental feasibility of the proposed KRu_4_Z_12_ (Z = As, Sb) compound involves an evaluation of the formation energy per formula unit cell (*E*_For_) and the cohesive energy (*E*_Coh_) in the subsequent stage.^33^ The equations utilized for these computations are as follows:
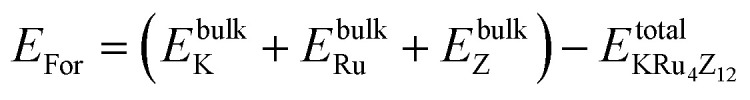

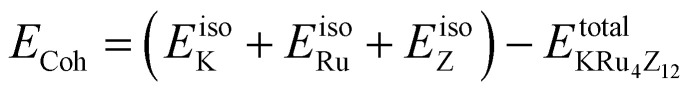


In this context, *E*_For_ represents the energy associated with the KRu_4_Z_12_ (Z = As, Sb) molecule or formula unit cell. *E*^bulk^_K_, *E*^bulk^_Ru_, and *E*^bulk^_Z_denote the energies of individual K, Ru, As, and Sb atoms in a bulk state, respectively. The energies of free space atoms are used to estimate these values. *E*^iso^_K_, *E*^iso^_Ru_ and *E*^iso^_Z_ (Z = As, Sb) represent the isolated atomic energies of the constituent atoms. Furthermore, the total energy per formula unit of the KRu_4_X_12_ (X = As, Sb) compound is represented as 
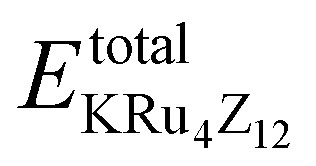
. The negative value of the formation energy (−0.25 and −0.22 eV per atom) for KRu_4_Z_12_ (Z = As, Sb) suggests its stability and the potential for experimental synthesis. Additionally, the cohesive energy (*E*_Coh_) reflects the strength of bonding among the components. The substantial magnitude of *E*_Coh_ (4.07 and 3.57 eV per atom) highlights remarkable chemical stability and the formation of robust bonds between the constituent atoms within the KRu_4_Z_12_ (Z = As, Sb) molecule.

By employing the provided formula, the enthalpy of formation energy (denoted as Δ*E*) is utilized to evaluate the stability of the compounds.^34^ The calculation is represented as follows:Δ*E* = *E*_total_ − *aE*_A_ − *bE*_B_ − *XE*_X_here, *E*_total_ signifies the overall energy of KRu_4_X_12_ (X = As, Sb), where K, Ru, and X are represented by *E*_A_, *E*_B_, and *E*_X_ respectively. The computed energy values for KRu_4_X_12_ (X = As, Sb) are −5.03 Ry, and −4.46 Ry respectively. These values indicate that the negative enthalpy of formation energy reinforces the stability of the compounds.

### 3.2 Electronic properties

**Fig. 3:**
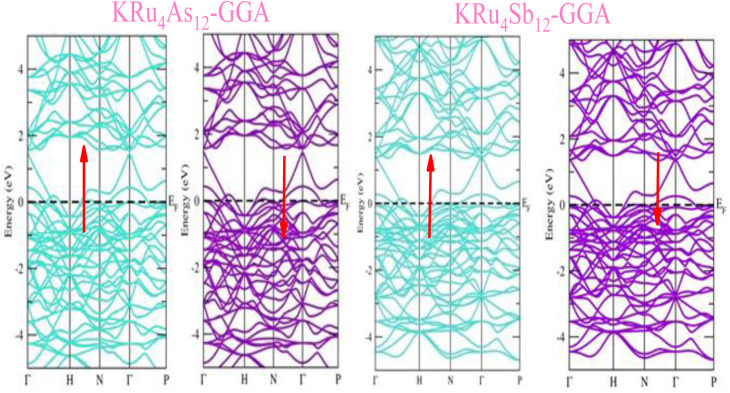
These are the band structures of KRu_4_As_12_ and KRu_4_Sb_12_ within the schemes of the GGA method. The arrows indicating spin channels, representing spin-up (↑) and spin-down (↓).

**Fig. 4:**
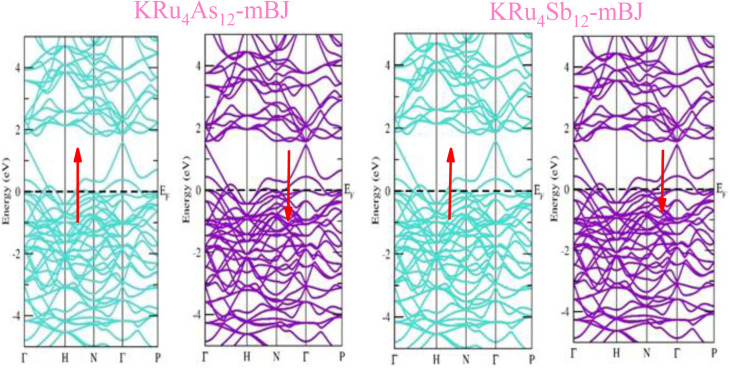
These are the band structures of KRu_4_As_12_ and KRu_4_Sb_12_ within the schemes of GGA and GGA + mBJ methods. The arrows indicating spin channels representing, spin-up (↑) and spin-down (↓).

**Fig. 5:**
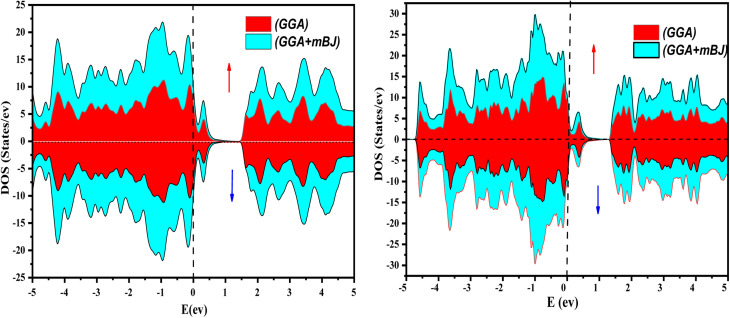
The total density of states of KRu_4_As_12_ and KRu_4_Sb_12_*via* GGA and GGA + mBJ.

The electronic properties of both KRu_4_As_12_ and KRu_4_Sb_12_ have been investigated by analyzing their band structures and density of states (DOS). However, a thorough discussion of the electronic properties of any material plays a significant role in realizing its advantages in various research areas. In addition, the two-dimensional band structures and density of states (DOS) of the material provide essential information about the electrical properties of the material. In the present investigation, we have used GGA and GGA + mBJ approximation to predict the electronic properties. Fig. 3 and 4 show the spin-polarized band structures using the GGA and GGA + mBJ approximations. It can be observed that the energy bands in both the spin channels cross over the Fermi level (*E*_F_), indicating the metallic character. Additionally, implementing the potential over GGA describes the comparative shift in energy states due to including potential over GGA + mBJ (Fig. 5).

**Fig. 6:**
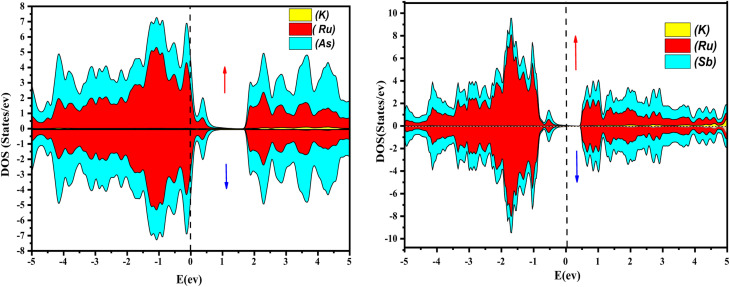
Atomic contribution towards band formation of KRu_4_As_12_ and KRu_4_Sb_12_ by GGA + mBJ method.

**Fig. 7:**
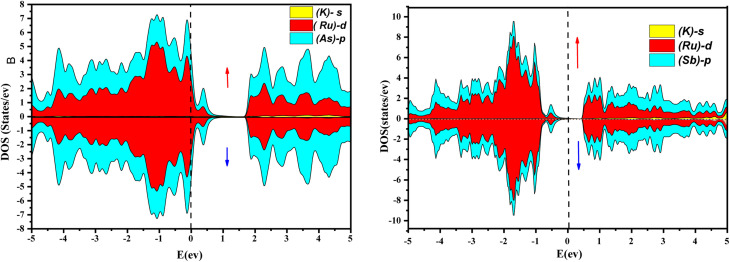
Orbital disintegrated density of state of KRu_4_As_12_ and KRu_4_Sb_12_ by GGA + mBJ method.

Further interpretation can be conducted by analyzing the density of states. The metallic character is also reflected in the TDOS plots for both spin orientations of KRu_4_As_12_ and KRu_4_Sb_12_. The geometric plotting in Fig. 6 and 7 illustrates that ruthenium (Ru) is more competent in describing electronic properties. The elements (arsenic, antimony) X-p states play a minor role in this conductor behavior but are primarily dominant in Ru-d orbitals. In the range of −5 to 5 eV, the Ru-d states and X-p states are dominant, and the Ru-d states strongly hybridize with the X-p states. Since potassium (K)-s states do not exist near the Fermi level, they provide minor information about the electronic structure. Below the Fermi level, the Ru-d states dominate, while above the Fermi level, the X-p states dominate.^46^

**Fig. 8:**
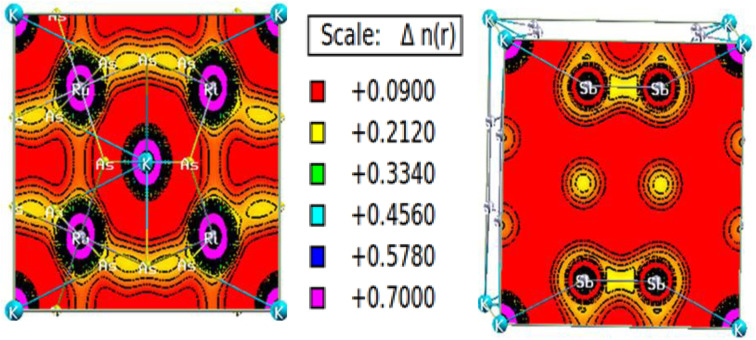
Density of spin-polarized electrons along (111) for bcc KRu_4_As_12_ and (001) KRu_4_Sb_12_ filled skutterudite.

The charge density calculation is often presented in a plane, where it tells us the direction of the charge transfer and nature of bonding in the material, namely, whether it is the ionic or covalent type of bond. The charged density plots are typically used to analyze electrons accumulating around atoms. It is challenging to analyze bonding if there is significant charge accumulation between two atoms. Still, if the contour around each atom is not symmetric, there will be a complex type of interaction. Fig. 8 presents the two-dimensional (2-D) electron charge distributions for KRu_4_As_12_ and KRu_4_Sb_12_ in the (111) and (001) planes. The colour differences around the atoms clearly show charge shearing in both plots. From the interpretation of charges, the description of electronic charge density provides a better understanding of chemical bonding. However, it considers non-bonding states and delivers overall charge density inside the material. Compared with d-group elements, the transition metal exhibits the maximum contribution on the two-electron density plots. As the density plots for transition metals show, transition metals have the most significant contribution, whereas p-group elements have the least. Typically, both the alloys KRu_4_As_12_ and KRu_4_Sb_12_ exhibit ionic bonding characteristics, while the bonding between the transition metals and Sb appears to be covalent. Therefore, the illustration of the electron charge density graph suggests that these skutterudite materials preserve both ionic and covalent bonds.^47^

### 3.3 Mechanical properties

Mechanical properties are defined as a material's physical properties that are exhibited when forces are applied. These properties include the modulus of elasticity, tensile strength, hardness, and fatigue limit. These properties are important because they describe the material's compressibility, strength, ductility, brittleness, *etc.* In order to define these properties, we evaluated the elastic constants of KRu_4_As_12_ and KRu_4_Sb_12_ using the cubic elastic code developed by Murtaza Jamal.^18^ The elastic parameters obtained from this evaluation are listed in Table 2. Due to the highly cubic symmetry of KRu_4_As_12_ and KRu_4_Sb_12_, only three independent elastic constants are required, namely *C*_11_, *C*_12_, and *C*_44_. Using Born–Huang stability criteria condition:^48,49^*C*_11_ – *C*_12_ > 0; *C*_44_ > 0; +2*C*_12_ > 0

**Table 2:** Calculations of different elastic parameters *C*_11_, *C*_12_, *C*_44_ in GPa, bulk modulus (*B* in GPa), shear modulus (*G* in GPa), Young modulus (*Y* in GPa), Pugh's ratio (*B*/*G*), Cauchy pressure (*C*_p_ in GPa), anisotropy factor (*A*), Poisson's ratio (*ν*), melting temperature (*T*_m_ in K) at 0 GPa and 0 K for KRu_4_As_12_ and KRu_4_Sb_12_ compounds.

Parameter	*C*_11_	*C*_12_	*C*_44_	*B*	*G*	*Y*	*B*/*G*	*C*_p_	*A*	*ν*	*T*_m_ (K)
KRu_4_As_12_	184.71	82.79	46.75	116.76	48.35	127.56	2.41	63.04	0.19	0.31	1644.83
KRu_4_Sb_12_	175.87	42.80	80.09	87.16	74.36	173.69	1.17	−37.28	1.20	0.16	1592.62
LiFe_4_As_12_ (ref. 17)	214.79	65.77	51.64	115.44	59.82	153.04	1.92	14.13	0.69	0.28	1822.62
LiFe_4_Sb_12_ (ref. 17)	206.43	53.01	47.49	104.14	57.60	145.90	1.80	5.53	0.61	0.27	1773.19
SrFe_4_Sb_12_ (ref. 37)	252.47	58.85	47.40	12.35	67.15	170.51	1.83	—	0.48	0.26	—
CoSb_3_ (ref. 42)	185.13	46.95	43.02	—	—	165.9	0.19	—	—	0.19	—

Furthermore, the mechanical properties of these skutterudites are affected by several additional parameters. Using the Voigt–Reuss–Hill scheme, one can determine the bulk modulus (*B*), shear modulus (*G*), and Young's modulus (*Y*) to predict the hardness, compressibility, and stiffness of a material. For the cubic system, the Voigt bounds for bulk modulus (*B*) and shear modulus (*G*) are calculated as:^50^
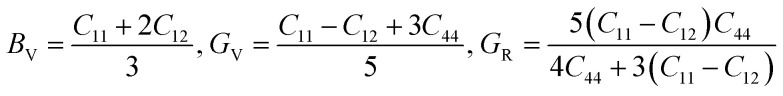


However, Hill defined that any material's *B* and *G* values should be averaged by Voigt and Reuss limits.^51^
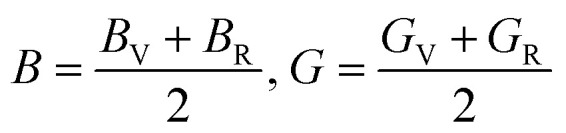


Young's modulus (*Y*) determines a material's strength, a ratio of linear stress and strain. The Young modulus (*Y*) defines the material's stiffness, while *B* means resistance to volumetric deformation, and *G* means resistance to shape deformation. Table 2 presents the calculated values of *B* and *G*.^52^ The Young's modulus and Poisson ratio can be determined through mathematical formulation:
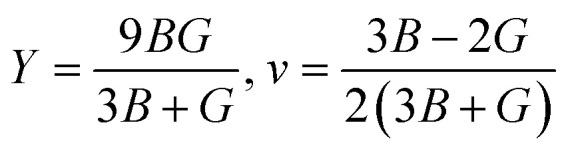


The calculated data in Table 2 show that *Y* > *B* > *G* indicates the stiffness of these skutterudite materials. We have calculated these mechanical parameters to define the ductility and brittleness of these alloys. Parameters like Pugh's ratio (*B*/*G*), Poisson's ratios, and Cauchy pressure are used to quantify these properties.^53^ If the value of *B*/*G* is more significant than 1.75, it indicates that the material is ductile or otherwise brittle. The *B*/*G* (ref. 53) value of KRu_4_Sb_12_ is 1.17, which is less than 1.75, meaning it is brittle. The *B*/*G* value of KRu_4_As_12_ is 2.57, which means ductile in nature. If the *C*_p_ value is positive, these materials are ductile; otherwise, they are brittle.^54^ A negative value of *C*_p_ supports the brittle nature of KRu4As_12_ and a positive value of *C*_p_ refers to the ductile nature of KRu_4_Sb_12_. We can also evaluate the Poisson's ratio (*ν*); if it is below 0.33, it is brittle; otherwise, it is ductile.^50^ The KRu_4_Sb_12_ presents a value of 0.16, indicating its brittleness, and KRu_4_As_12_ presents a value of 0.31, its brittle one. The material has an isotropic response whenever the anisotropic factor (*A*) equals unity. Otherwise, the material shows an anisotropic response. The degree of anisotropy increases as the deviation from unity increases. The calculated values of the anisotropic factor (*A*) for KRu_4_As_12_ and KRu_4_Sb_12_ are 0.91 and 1.20, indicating that the materials are anisotropic. The melting temperature (*T*_m_)^55^ can be calculated from elastic constants using the formula illustrated:*T*_m_ (K) = [553 + (5.911)*C*_11_] ± 300 K

The calculated melting temperature of KRu_4_As_12_ and KRu_4_Sb_12_ are 1644.83 K and 1592.62 K, respectively. The Debye temperature is calculated using the average sound velocity (*V*_m_).
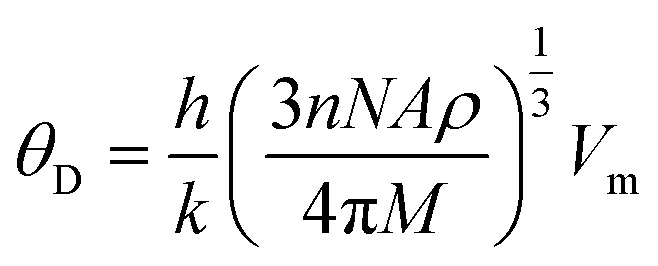
here, *h* and *k* are Plank's and Boltzmann's constants, respectively, and are given as the average sound velocity. The presented *V*_t_ and *V*_l_ are transverse and longitudinal velocities calculated using Navier's equation.^52^
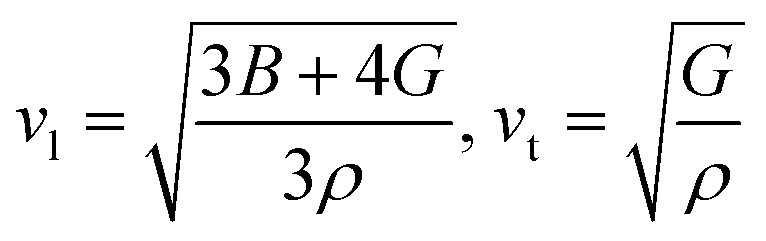


Both *V*_t_ and *V*_l_ present velocities differ along different planes, namely [100], [110], and [111]. There is a maximum longitudinal velocity of 4232 m s^−1^ along the [100] direction and a maximum transverse velocity of 3445 m s^−1^ along the [110] plane.^56^Table 3 presents the calculated values of *V*_l_, *V*_m_, *V*_t_, and Debye temperatures.

**Table 3:** Velocities of directional elastic waves and the Debye temperature of KRu_4_As_12_ and KRu_4_Sb_12_ skutterudite

Materials	[100]		[110]		[111]		Average velocities			*θ*_D_ (K)
	*V*_l_	*V*_t_	*V*_l_	*V*_t_	*V*_l_	*V*_t_	*V*_l_	*V*_t_	*V*_m_	
KRu_4_As_12_	4232	3143	3903	2129	4167	2129	4.19	2.16	2.42	231
KRu_4_Sb_12_	3960	3445	3650	2672	4159	2672	4.07	2.57	2.83	678

### 3.4 Thermoelectric properties

Boltzmann's theory has been applied to study the thermoelectric properties of KRu_4_As_12_ and KRu_4_Sb_12_ materials. The thermoelectric parameters were obtained using the BoltzTraP code, which is embedded in the Wien2k simulation package to solve the Boltzmann transport equation.^57–62^ These properties include electrical conductivity (*σ*/*k*), electrical and thermal conductivity (*κ*_e_), Seebeck coefficient (*S*), and power factor (PF) under constant relaxation time approximations. The maximum conversion efficiency of the TE device is expressible as;^14^
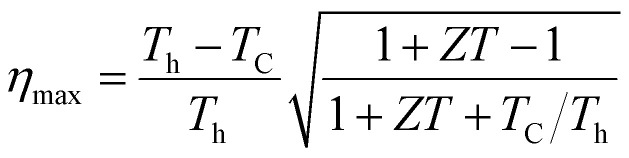


In thermoelectric (TE) systems, *T*_h_ and *T*_c_ denote the temperatures of the hot and cold sides, respectively, while *ZT* represents the figure-of-merit of the TE material. The conversion efficiency of TE devices improves with increasing *ZT*, making the development of both p-type and n-type materials with high *ZT* essential for creating efficient TE devices. It's important to note that besides *ZT*, the efficiency of thermoelectric generators (TEGs) also relies on selecting an optimal contact material.

#### 3.4.1 Seebeck coefficient (*S*)

**Fig. 9:**
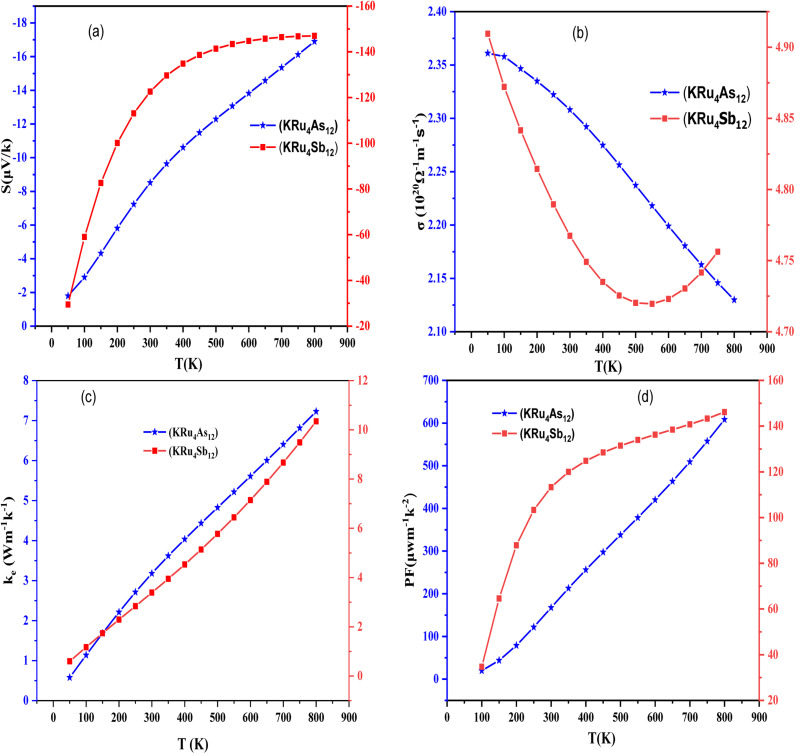
Temperature-dependent properties of KRu_4_As_12_ and KRu_4_Sb_12_ materials. (a) Seebeck coefficient, (b) electrical conductivity, (c) electronic thermal conductivity and (d) power factor are plotted as functions of temperature for both materials.

The thermoelectric phenomenon in materials can be quantified using various parameters, among them the Seebeck coefficient, which is a vital parameter due to its voltage sensitivity for a given temperature gradient. Generally, a high Seebeck coefficient leads to good thermoelectric properties of materials. The Seebeck coefficient provides a sensitive measure of the electronic structure near Fermi energy. If the Seebeck coefficient is negative, it indicates that electrons are the majority carriers. The temperature dependence of *S* for the temperature range of 50 K to 800 K can be illustrated in Fig. 9(a). At room temperature (300 K); *S* acquires a value of 9 µV K^−1^ for KRu_4_As_12_ and 14 µV K^−1^ for KRu_4_Sb_12_. At 800 K the value of *S* is 16 µV K^−1^ for KRu_4_As_12_ and 15 µV K^−1^ for KRu_4_Sb_12_.

#### 3.4.2 Electrical conductivity (*σ*/*τ*)

Electrical conductivity is also an important aspect of defining the transport properties. It is a measure of how easily charge carriers pass through a material and can be denoted as (*σ*/*τ*). The Wiedemann–Franz law is used to determine the conductivity. As shown in Fig. 9(b), the electrical conductivities of filled skutterudites KRu_4_As_12_ and KRu_4_Sb_12_ are temperature-dependent as illustrated in Fig. 9(b). The electrical conductivities over relaxation time (*σ*/*τ*) of the present investigated compounds decreased with increasing temperature. This means that the filler atoms contribute to the reduction of electrical resistivities. The electrical conductivity of these compounds is of the order of 10^20^ Ω^−1^ m^−1^ s^−1^. The calculated value of electrical conductivity at 50 K for KRu_4_As_12_ is 2.36 × 10^20^ Ω^−1^ m^−1^ s^−1^ and then decreased linearly to 2.31 × 10^20^ Ω^−1^ m^−1^ s^−1^ at room temperature. As a result, the electrical conductivity decreased gradually until it reached a peak of 2.10 × 10^20^ Ω^−1^ m^−1^ s^−1^ at 800 K for KRu_4_As_12_. At 50 K, the electrical conductivity of KRu_4_Sb_12_ is 4.90 × 10^20^ Ω^−1^ m^−1^ s^−1^ and reached 4.78× 10^20^ Ω^−1^ m^−1^ s^−1^ at 300 K. At 800 K; the electrical conductivity decreased continuously up to 4.75 ×10^20^ Ω^−1^ m^−1^ s^−1^.

#### 3.4.3 Electronic thermal conductivity (*κ*_e_)

To calculate thermal conductivity, we sum electronic and lattice parts by *κ* = *κ*_e_ + *κ*_l_. Where *κ*_e_ and *κ*_l_ represent the electronic and vibration components, respectively. Only the electronic contribution of thermal conductivity has been considered in this study since BoltzTraP can only calculate the electronic part *κ*_e_ of thermal conductivity. In Fig. 9(c), we present the electronic thermal conductivity of the KRu_4_As_12_ and KRu_4_Sb_12_ metallic skutterudites. From the graphical plot of thermal conductivity, *κ*_e_ increases linearly with temperature mainly due to the thermal energy of the electrons.

#### 3.4.4 Power factor (*S*^2^*σ*)

Power factor is a measure the thermoelectric efficiency of a compound. It is calculated using the equation PF = *S*^2^*σ*, where *S* is the Seebeck coefficient, and *σ* represents the material's electrical conductivity. The calculated power factors for the KRu_4_As_12_ and KRu_4_Sb_12_ skutterudite compounds are shown in Fig. 9(d). These materials demonstrate their suitability for high-temperature applications as the power factor increases with rising temperature. At the maximum, the power factor reaches 608.43 µW m^−1^ K^−2^ for KRu_4_As_12_ and KRu_4_Sb_12_ 146.16 µW m^−1^ K^−2^. Calculation of different transport parameters at 300 K for KRu_4_As_12_ and KRu_4_Sb_12_ as represented in Table 4.

**Table 4:** Calculation of different transport parameters at 300 K for KRu_4_As_12_ and KRu_4_Sb_12_

Material	Seebeck coefficient (µV K^−1^)	Electrical conductivity (Ω^−1^ m^−1^ s^−1^)	Thermal conductivity (W m^−1^ K^−1^)	Power factor (µW m^−1^ K^−2^)
KRu_4_As_12_	−8.52082	2.30805	1.62826	167.5441
KRu_4_As_12_	−122.592	4.78953	3.38709	113.28731
LiFe_4_As_12_ (ref. 17)	5	2.6	1.8	0
LiFe_4_Sb_12_ (ref. 17)	15	2.6	5.1	150
SrFe_4_As_12_ (ref. 40)	−120	0.56	0.8	0.7 (W m^−1^ K^−2^ s^−1^)
CoSb_3_ (ref. 43–45)	50; 0; 72	4; 0; 0.429	2; 0; 117	—

### 3.5 Thermodynamic properties

**Fig. 10:**
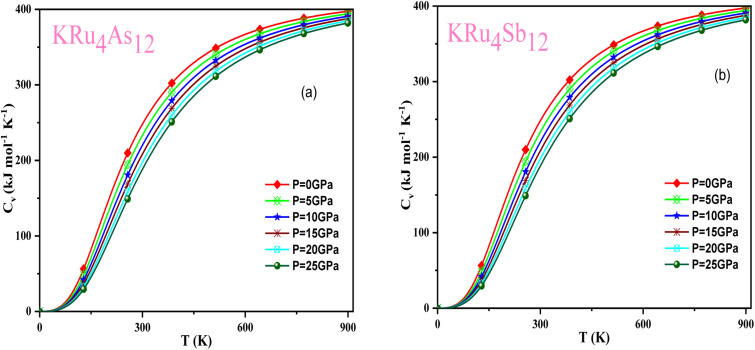
Variation of *C*_V_ with temperature at different pressure for (a) KRu_4_As_12_ (b) KRu_4_Sb_12_materials.

Based on the Gibbs2 code and a quasi-harmonic Debye model,^63,64^ we determined the thermodynamic parameters of the filled skutterudite materials KRu_4_As_12_ and KRu_4_Sb_12_ under high pressure and temperature. In the present investigation, a set of total energy calculations *versus* unit cell volume (*E*–*V*) in the static approximation was performed. Afterward, we applied Murnaghan's EOS to determine the structural parameters at zero pressure and temperature. Subsequently, we computed the macroscopic properties based on standard thermodynamic relations. Temperatures ranging between 0 and 900 K are used to determine the thermal properties. This study examines pressure effects ranging from 0 to 25 GPa. Specific heat capacity (*C*_V_) helps understand heat movement in crystals, lattice vibrations, or phase transfer processes. A plot of the heat capacity *C*_V_*versus* temperature at 0 to 25 GPa is shown in Fig. 10. From the figure, it is observed that *C*_V_ values increase rapidly at low temperatures, then increase gradually at high temperatures, and follow Dulong Petit limits,^58^ which are common characteristics for all solids at high temperatures. The increase in *C*_V_ with temperature is primarily attributed to the rise in atomic vibrations, as shown in Fig. 10. Furthermore, the *C*_V_ follows the Debye T3 law at the steep low-temperature slope.^65^ At high temperatures, *C*_V_ approaches 409.617 kJ mol^−1^ K^−1^ and 400 kJ mol^−1^ K^−1^ for KRu_4_As_12_ and KRu_4_Sb_12_, respectively. Based on the calculations at zero pressure and 300 K, the calculated value of *C*_V_ are 310.08 kJ mol^−1^ K^−1^ for KRu_4_As_12_ and 734.58 kJ mol^−1^ K^−1^ for KRu_4_Sb_12_ materials.

**Fig. 11:**
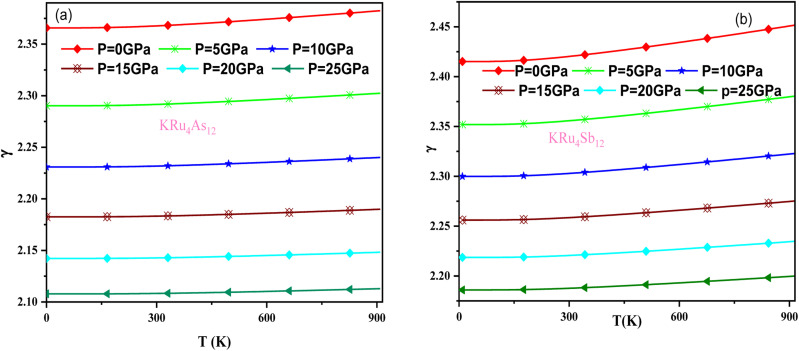
Variation of Grüneisen parameter with temperature and pressure for (a) KRu4As_12_ (b) KRu_4_Sb_12_materials.

The effect of pressure and temperature on the Grüneisen parameter (*γ*) has been studied and is shown in Fig. 11. The Grüneisen parameter (*γ*) is an important thermodynamic property widely used to predict thermoelastic behavior in solids. It delivers the concept of anharmonicity in solids, and various physical properties such as bulk modulus, specific heat, and frequency of lattice vibrations are directly related to it. When examining the temperature rise, a slow increase in *γ* can be observed, suggesting the presence of anharmonic effects.

**Fig. 12:**
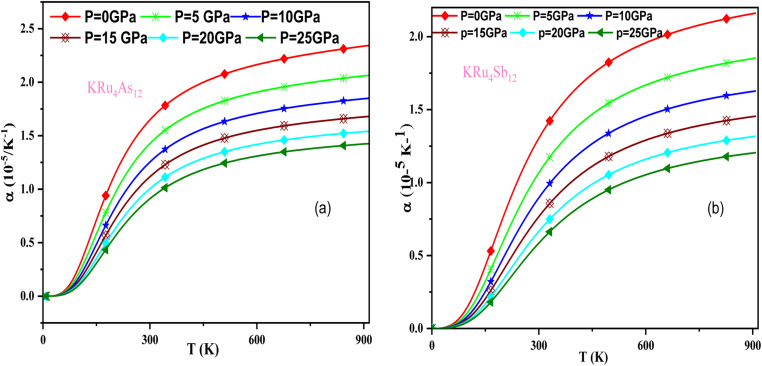
The variation of thermal expansion with temperature and pressure for (a) KRu_4_As_12_ (b) KRu_4_ Sb_12_materials.

**Fig. 13:**
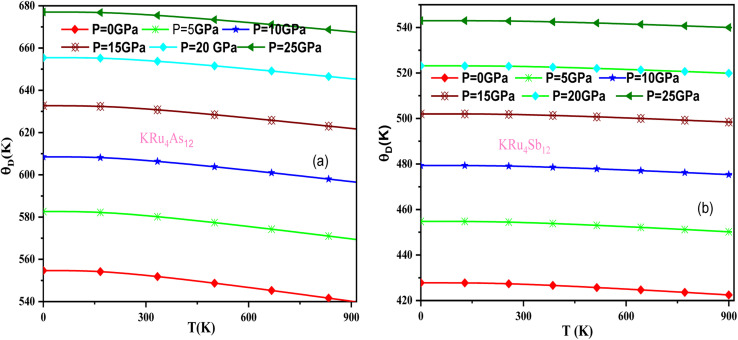
Variation of Debye temperature with pressure and temperature T(K). (a) KRu_4_As_12_ (b) KRu_4_Sb_12_ materials.

In these materials, the variation in temperature is noticeable with the application of the pressure, *γ* decreases significantly, as seen in the graphical plot.^66^Fig. 12 shows our examination of the effect of temperature and pressure on thermal expansion, further contributing to our understanding of the materials' behavior. We find that the thermal expansion coefficient increases as temperature increases for both compounds. At a given temperature, the thermal expansion coefficient *α* decreases rapidly as pressure increases and becomes smaller at higher temperatures and pressures. In a given pressure range, *α* increases rapidly with increasing temperature up to 150 degrees Celsius above the reference temperature. Above this threshold, *α* approaches linear behavior with further increases in temperature. Considering a pressure of zero and a temperature of 300 K, the thermal expansion valve is 1.63 × 10^−5^ K^−1^ for KRu_4_As_12_ and 2.20 × 10^−5^ K^−1^ for KRu_4_Sb_12_. The crystals behave classically above this temperature because thermal vibrations become more significant than quantum effects. The variation of the Debye temperature as a function of pressure and temperature is shown in Fig. 13. It can be seen that *θ*_D_ remains almost constant from 0 to 150 K. However, above this temperature, *θ*_D_ exhibits a regular and linear decrease with increasing temperature. For a given temperature, the Debye temperature increases almost linearly. The calculated value of *θ*_D_ at zero pressure and zero temperature is 554.68 K for KRu_4_As_12_ and 427.72 K for KRu_4_Sb_12_ compounds. Table 5: the calculated value of thermal parameters at 300 K for different pressure range (0–25).

**Table 5:** The calculated value of thermal parameters at 300 K for different pressure range (0–25)

Pressure (GPa)	*C*_V_ (kJ mol^−1^)	*γ*	*α* (10^−5^ K^−1^)	*θ*_D_ (K)
**KRu**_**4**_ **As**_**12**_ **300 K**				
0	375.6823	2.20693	3.05355	470.92
5	362.6941	2.06396	2.20711	537.03
10	351.1887	1.97567	1.73754	591.9
15	340.7492	1.91419	1.43534	639.62
20	331.1397	1.86818	1.22303	682.3
25	322.2085	1.83207	1.06493	721.2

**KRu**_**4**_ **Sb**_**12**_ **300 K**				
0	358.5945	5.94429	2.94496	559.28
5	338.7098	5.05083	1.68895	651.26
10	323.1228	4.58198	1.18278	719.9
10	309.9896	4.27858	0.90682	776.4
20	298.5178	4.06055	0.90682	825.21
25	288.2753	3.89356	0.61215	868.63

**LiFe**_**4**_ **As**_**12**_ **(ref. 17) 300 K**				
0	358.06	2.25	2.22	561.04
5	351.81	2.18	1.95	590.67
10	345.91	2.14	1.73	617.98
15	340.31	2.09	1.56	643.42
20	334.97	2.06	1.42	667.30
25	329.86	2.03	1.30	689.86

**LiFe**_**4**_ **Sb**_**12**_ **(ref. 17) at 300 K**				
0	385.42	2.17	2.24	421.28
5	380.52	2.09	1.91	449.23
10	375.92	2.03	1.66	474.42
15	371.56	1.98	1.48	497.50
20	367.40	1.93	1.34	518.89
25	363.42	1.90	1.22	538.89

**CoSb**_**3**_ **(ref. 44) at 300 K**				
0	—	0.952	6.36	307

## 4. Conclusion

The structural, mechanical, electronic, thermal, and thermoelectric properties of filled skutterudite materials, specifically KRu_4_As_12_ and KRu_4_Sb_12_, were thoroughly investigated using the GGA and GGA + mBJ potentials. The calculated structural properties, including lattice parameter, bulk modulus, and energy, are compared with available theoretical data. The evaluated elastic parameters reflect that these alloys are mechanically stable. The hardness of these compounds can be defined by considering the dependence of pressure on the elastic constant. The calculated data also revealed that KRu_4_As_12_ behaves as a brittle material, while KRu_4_Sb_12_ acts as a ductile material. By using spin-polarized band structure and DOS plots, KRu_4_As_12_ and KRu4Sb12 are found to exhibit metallic behavior. Furthermore, the calculated thermal and thermoelectric parameters indicate that these materials are thermodynamically stable and show potential for thermoelectric applications. This represents a significant finding, as it is the first-ever observation of such behavior in these compounds. Consequently, the calculated values provide a promising route for experimentalists to synthesize these materials successfully.

## Conflicts of interest

There are no conflicts to declare.

## Supplementary Material
