# Correction: Theoretical exploration of inherent electronic, structural, mechanical, thermoelectric, and thermophysical response of KRu_4_Z_12_ (Z = As_12_, Sb_12_) filled skutterudite materials

**DOI:** 10.1039/d3ra90097e

**Published:** 2023-09-28

**Authors:** Poorva Nayak, Pankaj Srivastava, Dinesh C. Gupta

**Affiliations:** a Condensed Matter Theory Group, School of Studies in Physics, Jiwaji University Gwalior 474 011 India poorvanayak11@gmail.com sosfizix@gmail.com; b Atal Bihari Vajpayee Indian Institute of Information Technology and Management Gwalior 474015 India

## Abstract

Correction for ‘Theoretical exploration of inherent electronic, structural, mechanical, thermoelectric, and thermophysical response of KRu_4_Z_12_ (Z = As_12_, Sb_12_) filled skutterudite materials’ by Poorva Nayak *et al.*, *RSC Adv.*, 2023, **13**, 27873–27886, https://doi.org/10.1039/D3RA05546A.

The authors regret that the name of one of the authors (Pankaj Srivastava) was shown incorrectly in the original article. The corrected author list is as shown above.

The Royal Society of Chemistry apologises for these errors and any consequent inconvenience to authors and readers.

## Supplementary Material

